# Contribution to the Study of Septic Intoxication

**Published:** 1888-02

**Authors:** 


### *CONTRIBUTION TO THE STUDY OF SEPTIC INTOXICATION.*

The following is a summary of the conclusions reached by Koserowton in a paper on this interesting subject:

1. The microbes of putrefaction introduced into the tissues or blood of healthy animals induces no pathological change.

2. The saline solution in which the microbes of putrefaction are developed changes its properties and becomes a true poison.

3. The poisonous principles found in this saline solution resemble none of the products which may be obtained from the salts, and, consequently, must be produced or excreted by the microbes, the saline solution serving as a culture fluid.

4. When these putrefactive processes take place in the presence of air, chemical compounds soluble in water and alcohol are developed, which, on injection in animals, will produce elevation of temperature.

5. Putrefactive processes, with the air excluded, form chemical compounds, which act principally on the nervous system.

6. Inoculation with putrid matter is followed by symptoms differing materially from those of an infectious disease, and corresponding closely with those of poisoning.

7. The active principle of putrid matter is a chemical compound, the action of which depends upon the relative amount or dose given to the animal experimented on.—*Wratsh.*

